# Does Guilt Help or Hinder Gratitude? Personal Distress, Guilt Proneness, and Gender Differences in Adolescents

**DOI:** 10.3390/children13040539

**Published:** 2026-04-13

**Authors:** Sepideh Yasiniyan, Sandra Bosacki, Victoria Talwar

**Affiliations:** 1 Faculty of Educational and Counselling Psychology, McGill University, Montreal, QC H3A 0G4, Canada; 2 Faculty of Education, Brock University, 1812 Sir Isaac Brock Way, St. Catharines, ON L2S 3A1, Canada

**Keywords:** gratitude, personal distress, guilt proneness, moral emotions, adolescence

## Abstract

**Highlights:**

**What are the main findings?**

- Personal distress at Time 1 was associated with lower gratitude at Time 2 at moderate to high levels of guilt proneness, but not at low levels of guilt proneness.
- Gender did not significantly moderate the association between personal distress and gratitude.

**What are the implications of the main findings?**

- Lower gratitude in adolescence may reflect the joint role of personal distress and guilt proneness rather than ingratitude itself.
- Guilt proneness should be considered when studying adolescents’ gratitude and socioemotional development.

**Abstract:**

Background: Adolescents are at an increased risk for experiencing emotional reactions and interpersonal stressors, which can interfere with their access to gratitude. While gratitude is typically defined as an empathic or other-oriented emotion, personal distress is an aversive or self-oriented empathic reaction to others’ emotions or states, which can interfere with prosocial behavior. The goal of this study was to examine whether guilt proneness and gender moderate the prospective association between personal distress and later gratitude. Methods: The participants consisted of 111 early adolescents (61% females; M age = 12.74). Trait gratitude, personal distress (IRI—Personal Distress), and guilt proneness (TOSCA-A) were used as self-report measures. Using conditional process analysis (PROCESS Model 2), we tested whether Time 1 personal distress is associated with Time 2 gratitude, moderated by guilt and gender. Correlations showed that Time 2 gratitude was positively related to guilt but was not significantly related to personal distress. Results: The results indicated that personal distress was associated with lower Time 2 gratitude when guilt proneness was moderate to high, but not when guilt proneness was low. The association between personal distress and gratitude varied across levels of guilt proneness. Although conditional effects were examined separately for boys and girls, the interaction with gender was not significant and should be interpreted cautiously. The findings suggest that lower gratitude in adolescence may reflect distress–guilt dynamics rather than ingratitude itself. Conclusions: These findings highlight the importance of considering guilt proneness in future research on adolescents’ socioemotional development.

## 1. Introduction

Adolescents often experience high levels of emotional reactivity and susceptibility to interpersonal stressors, which may escalate to personal distress. Such feelings of discomfort are in response to others’ suffering or perceived sacrifices, which can evoke negative emotions combined with a sense of responsibility. Empathy is a multidimensional and multifaceted construct [1,2,3]. It includes cognitive and affective components that affect the capacity to understand and internalize others’ emotional experiences. The components are correlated, and one can be aware of the emotional states of the other without necessitating emotional resonance. Empathy can also take the form of other- or self-oriented responses. The former includes empathic concern (being aware of the emotional state of others) while the latter comprises personal distress that can automatically manifest in a shutdown due to emotional overload [4,5]. Several studies have tested empathy as a determinant of prosocial behavior. Initial reports show that different responses to emotional states serve different functions in directing or deterring helping behaviors, with sympathy being other-oriented while personal distress is self-oriented [6,7,8].

For example, when adolescents consider what they have and the sacrifices their parents have made for them, they may experience feelings of sorrow for their parents or a personal responsibility to repay their sacrifices [9,10]. Instead of encouraging feelings of gratitude, these reflections may instill feelings of guilt and emotional distress, especially if the adolescents feel that they cannot repay their parents’ sacrifices [11,12]. A similar state of emotional tension can also be experienced by adolescents if they have a sibling with a disability. They may be aware of their own advantages and feel grateful, but seeing their sibling suffer can increase their personal distress and feelings of guilt, making it difficult for them to feel gratitude. In each of these examples, the adolescent’s response can be described as personal distress elicited by another’s suffering. More broadly, although empathy is often linked to prosocial responding, research suggests that different components of empathy may lead to different outcomes. For example, experimental findings show that helping behavior is reduced when personal distress outweighs empathic concern, indicating that self-focused emotional responses may inhibit prosocial action [13].

Building on the prior literature, the present study focuses specifically on how internal emotional experiences—particularly the interplay between empathy and personal distress—may shape adolescents’ capacity to experience gratitude in response to others’ suffering. Although previous studies have found that distress and guilt are related to decreases in positive affect, little is known about how these experiences are associated with later gratitude during adolescence [14,15]. Gratitude is often characterized as a prosocial orientation, and one of the interpersonal-oriented moral emotions [16,17]. Studies on how empathy-related processes may inhibit or enhance gratitude reveal more about how the socioemotional infrastructure plays a role in moral growth during adolescence [18].

The present study conceptualizes guilt proneness as a dispositional tendency that shapes how adolescents interpret distress in interpersonal contexts, rather than as a concurrent state response. Personal distress and guilt proneness are expected to interact such that adolescents higher in guilt proneness are more likely to interpret distress in terms of self-blame, obligation, or indebtedness, thereby constraining gratitude. The present study addresses this gap by examining whether personal distress and guilt proneness are prospectively associated with later gratitude.

Gratitude has been described as a moral emotion arising when people feel that they are recipients of the deliberate actions of others [16,17]. In the psychological literature, gratitude is often considered to be an interpersonal emotion directed toward a benefactor [18]. However, broader conceptualizations extend beyond interpersonal exchanges to include a general sense of appreciation for positive outcomes, including those attributed to external sources such as nature, fate, or life circumstances [16,17,19]. In the present study, gratitude is conceptualized as a broad dispositional construct that includes both interpersonal appreciation and a general appreciation of positive life circumstances. Therefore, gratitude can be understood as reflecting an individual’s overall orientation toward recognizing and valuing positive experiences. Importantly, receiving benefits—whether from others or from broader life circumstances—may also evoke feelings of indebtedness or obligation, particularly when individuals feel pressured to reciprocate or view themselves as undeserving [11,20,21]. In these cases, gratitude may be accompanied by negative emotions, as self-blame and perceived obligation can constrain the experience of appreciation [22,23,24].

Guilt is also a self-conscious moral emotion associated with perceived responsibility for harming others, or the belief that one fails to meet interpersonal or moral obligations [14,25,26]. Although guilt may co-occur with emotional distress, it is conceptually distinct from personal distress because it involves moral self-evaluation and responsibility attribution [14,27,28]. Personal distress and guilt proneness are related, but distinct. Personal distress refers to a self-focused aversive reaction to others’ suffering, whereas guilt involves moral self-evaluation and responsibility attribution. Guilt proneness refers to a relatively stable tendency to respond with guilt across situations [29,30].

Receiving a favor can evoke feelings of guilt, especially when an individual feels unable to repay the favor or feels indebted to the person who showed kindness [31,32,33,34,35]. In such cases, gratitude is an emotional reaction to a perceived benevolent act, while guilt is a response to a sense of responsibility. Studies suggest that people experience guilt as a consequence of failing to meet obligations to others [36]. Guilt inspires reparative behavior [14]. When guilt is high, adolescents view their own relief, support, and benefits as undeserved, and their obligations as a burden that detracts from gratitude [32,33,37]. Guilt proneness may influence how adolescents interpret distress in interpersonal contexts. Adolescents high in guilt proneness may be more likely associate distress with self-blame, perceived responsibility, or indebtedness, which may constrain their ability to experience gratitude. The emotional significance of receiving support may not be stable and may be perceived as appreciative or burdensome according to the emotional states of adolescents. Personal distress may heighten emotional arousal, while guilt may supply a moral interpretation that affects gratitude negatively as obligation to repay increases [32,37].

These processes suggest a potential mechanism whereby personal distress provides the emotional arousal, while guilt proneness shapes its interpretation, determining whether the experience facilitates appreciation or constrains gratitude. Despite its theoretical importance, no previous study has examined whether guilt proneness moderates the prospective association between personal distress and gratitude in adolescence. Specifically, it remains unclear whether personal distress is associated with lower subsequent gratitude particularly when adolescents are high in guilt proneness.

Gender may influence how adolescents interpret and respond to personal distress, particularly in relation to feelings of guilt and gratitude. These differences become more noticeable during adolescence, when girls generally report higher levels of guilt than boys, especially in situations involving harm to others or relationship problems [38]. This pattern is often explained by differences in socialization: girls are more likely to take responsibility for negative events, blame themselves, and dwell on their mistakes, whereas boys are more likely to minimize or deflect feelings of guilt, particularly in situations involving conflict or aggression. As a result, when experiencing personal distress, girls may be more likely to connect these feelings to a sense of personal responsibility, increasing the likelihood of guilt. Some prior work suggests that gratitude may be linked to somewhat different socioemotional processes in boys and girls, although such differences are typically modest and shaped by social context [39,40,41].

**Purpose of the present study**

The purpose of the current study is guided by the broaden and build theory of positive emotions [42,43]. Positive emotions broaden thought–action repertoires, while negative emotions like personal distress and guilt may constrict emotional openness and limit access to gratitude. Using a longitudinal design, the present study examines prospective associations between earlier emotional experiences and later gratitude, rather than developmental change.

The hypothesis was that personal distress would be associated with lower levels of subsequent gratitude, and that this would be moderated by guilt proneness. That is, personal distress would be positively or unrelated to gratitude at lower levels of guilt proneness but negatively associated with gratitude at higher levels of guilt proneness.

We also explored whether the association between personal distress and gratitude varied as a function of gender. Given mixed evidence and modest expected effects, gender differences are explored with caution as a secondary consideration rather than a primary explanatory mechanism. The literature shows that females experience greater guilt and empathic distress, and males experience less gratitude and fewer psychological benefits from it [39,44]. Girls also experience more empathic distress than boys [44,45].

## 2. Methods

### 2.1. Participants

At Time 1, the sample consisted of N = 113 participants (61% female; 39% male). At Time 1, participants ranged in age from 10.75 to 14.33 years (M = 12.74; SD = 0.99). At Time 2, data were available for 111 participants, with minimal attrition (1.8%). Given the minimal amount of missing data at follow-up (n = 2), analyses were conducted using complete cases. At follow-up, participants ranged in age from 12.08 to 15.08 years (M = 13.46; SD = 0.90). The parents of participants reported their level of education: the three most commonly held degrees were a bachelor’s degree (47.5%), college diploma (19%), and graduate degree (10%). Parents also reported their household income: the three most common were $100,000+ (41%), $80,000 to $99,000 (11%), and $70,000 to $80,000 (10%). Participants were primarily Canadian. Most of the participants were Caucasian, English speaking, and reflected predominately middle-class backgrounds.

This study received research ethics clearance from both the participating school boards and universities. This study draws on data from a larger 5-year longitudinal project examining adolescents’ social and emotional development. The present analyses focus on data collected at two consecutive waves (Time 1: 2015–2016; Time 2: 2016–2017) from students attending 13 schools in rural Canada. Participants were recruited from schools that agreed to participate following contact with school principals and approval from the relevant school authorities. All students in participating classrooms were eligible to participate if they returned signed parental/guardian consent and provided written assent. Students were excluded if parental consent or student assent was not provided on the day of testing. Participants were recruited from six schools (eight classes) from a medium-sized rural area in Eastern Canada. Participants were tested at Time 1 (T1) and 12 months later at Time 2 (T2). Data collection was conducted through pencil-and-paper self-report measures, which were administered either in the classroom or in a separate room (e.g., classroom or library) at the school.

### 2.2. Procedure

Participants were assessed at Time 1 and again 12 months later at Time 2. This interval was selected to capture short-term changes in adolescents’ emotional functioning during a period of heightened emotional reactivity and socio-emotional development, while remaining feasible within a school-based longitudinal design [46,47]. Although the study employed a longitudinal design, baseline levels of gratitude were not assessed at Time 1. As a result, the analyses examine prospective associations between earlier emotional characteristics and later gratitude rather than change over time.

### 2.3. Measures

#### 2.3.1. Gratitude, Resentment, and Appreciation Test (GRAT)

Trait gratitude was assessed using the Gratitude, Resentment, and Appreciation Test [33]. The original GRAT is a 44-item self-report measure with strong internal consistency (Cronbach’s α = 0.92) and demonstrated factorial validity, and it has been used in prior research with adolescent samples [31]. The measure comprises three subscales: a sense of abundance, simple appreciation, and an appreciation of others (social appreciation). In the present study, the short form of the GRAT was administered. This version includes 16 items (e.g., “I couldn’t have gotten where I am today without the help of many people”) rated on a five-point Likert scale ranging from one (strongly disagree) to five (strongly agree). The short GRAT has demonstrated good reliability and validity in previous research [31]. The GRAT has been widely used in adolescent and adult populations and is considered a robust measure of dispositional gratitude, capturing both interpersonal appreciation and broader life orientation toward positive experiences [17,31]. Internal consistency in the current sample was satisfactory (Cronbach’s α = 0.88). While the focus of the current research is on the interpersonal aspects of gratitude, the use of the complete GRAT measure allows for an assessment of both interpersonal and generalized gratitude, in line with the overall definition of gratitude.

#### 2.3.2. Personal Distress, Interpersonal Reactivity Index

The Interpersonal Reactivity Index (IRI) is a widely used self-report instrument designed to assess the multidimensional components of empathy [2]. It comprises four distinct subscales: fantasy (the tendency to imaginatively immerse oneself in fictional scenarios), perspective taking (the capacity to adopt others’ viewpoints), empathic concern (feelings of sympathy, warmth, and compassion for others), and personal distress (self-oriented feelings of anxiety, unease, or discomfort in response to others’ emotional suffering).

The full IRI includes 28 items, rated on a five-point Likert-type scale ranging from zero (“This statement does not describe me well”) to five (“This statement describes me very well”). An example item from the personal distress subscale is, “When I see someone who is hurt or upset, I feel overwhelmed with emotion.” In the present study, only the personal distress subscale was administered. This subscale was used to examine the degree to which participants experience aversive emotional reactions—such as anxiety or emotional discomfort—when witnessing the distress of others. Although the IRI includes multiple subscales, the present study focuses specifically on the personal distress component to isolate the self-oriented affective response to others’ suffering. This approach is consistent with theoretical and empirical work distinguishing personal distress from other-oriented empathic concern, with the former being more closely associated with emotional dysregulation and withdrawal rather than prosocial engagement [4,5]. Internal consistency for the personal distress subscale in the current sample was acceptable (Cronbach’s α = 0.70).

#### 2.3.3. Test of Self-Conscious Affect–Adolescent (TOSCA-A)

The TOSCA-A includes 15 scenarios (10 negative and 5 positive) that are likely events that could be experienced by adolescents [29]. Each scenario is followed by response items that assess guilt proneness and shame proneness. In the current study only the response items assessing guilt were used. An example of a scenario is “At lunchtime, you trip and spill your friend’s drink.” The guilty response is “I would feel very sorry. I should have watched where I was going.” For each scenario, adolescents rated the shame and guilt response items on a five-point scale (1 = very unlike me, 2 = a little unlike me, 3 = maybe (half and half), 4 = a little like me, and 5 = very like me) to indicate their likelihood of responding in the manner depicted. Although the TOSCA-A uses scenario-based prompts, it is widely conceptualized as a measure of guilt proneness because it captures individuals’ typical patterns of emotional responding across situations rather than momentary states [26,29]. Prior research supports the construct validity of this approach, showing that TOSCA-A guilt scores are associated with empathy and adaptive interpersonal functioning [17,48]. Internal consistency in the present sample was acceptable (Cronbach’s α = 0.79).

## 3. Results

The results of the correlation analysis (Table 1) indicate that gratitude at Time 2 was positively associated with guilt proneness at Time 1: r(109) = 0.48; *p* < 0.001. Gratitude was not significantly correlated with personal distress, with r(109) = −0.09 and *p* = 0.36, nor was personal distress significantly associated with guilt proneness, with r(109) = 0.12 and *p* = 0.20.

A regression model including personal distress, guilt proneness, and gender as predictors of Time 2 gratitude was conducted. The overall model was significant, with F(3, 107) = 12.67 and *p* < 0.001, explaining 26.2% of the variance in gratitude (R^2^ = 0.262). Guilt proneness significantly predicted higher levels of Time 2 gratitude: b = 0.48, SE = 0.09, t = 5.55, *p* < 0.001, and 95% CI [0.31, 0.65]. Personal distress was also a significant predictor, with b = −0.15, SE = 0.07, t = −2.01, *p* = 0.047, and 95% CI [−0.30, −0.002], indicating a small negative association with gratitude. Gender was not a significant predictor: b = −0.13, SE = 0.10, t = −1.24, *p* = 0.217, and 95% CI [−0.34, 0.08].

A moderated regression analysis using PROCESS Model 2 [49] examined whether guilt proneness and gender moderated the association between Time 1 personal distress and Time 2 gratitude. The overall model was significant, with F(5, 105) = 10.58 and *p* < 0.001, explaining 33.5% of the variance in gratitude (R^2^ = 0.33).

The interaction between personal distress and guilt was significant, with b = −0.38, SE = 0.12, t = −3.30, *p* = 0.001, 95% CI [−0.61, −0.15], ΔR^2^ = 0.069, F(1, 105) = 10.86, and *p* = 0.001, accounting for approximately 7% of additional variance in gratitude, reflecting a small-to-moderate effect size. This indicates that the association between personal distress and gratitude differed across levels of guilt proneness, such that higher levels of guilt strengthened the negative association between distress and later gratitude.

The interaction between personal distress and gender was not statistically significant, b = −0.29, SE = 0.16, t = −1.83, *p* = 0.070, and 95% CI [−0.60, 0.02]. This interaction accounted for a small and non-significant increase in explained variance (ΔR^2^ = 0.021) The full model explained 33.5% of the variance in gratitude (R^2^ = 0.335), indicating that the predictors accounted for a moderate proportion of variability in the outcome.

Accordingly, there was no evidence that the association between personal distress and gratitude differed as a function of gender. The combined inclusion of both interaction terms significantly improved model fit, ΔR^2^ = 0.073, F(2, 105) = 5.75, and *p* = 0.004. To probe the significant personal distress × guilt interaction, conditional effects of personal distress on gratitude were examined at low (16th percentile), medium (50th percentile), and high (84th percentile) levels of guilt, separately for females and males. At low levels of guilt, personal distress was not significantly associated with gratitude for either females, with b = 0.16, SE = 0.12, t = 1.35, and *p* = 0.179, or males, with b = −0.13, SE = 0.12, t = −1.02, and *p* = 0.311. At medium levels of guilt, personal distress was not significantly associated with gratitude for females, with b = −0.11, SE = 0.09, t = −1.26, and *p* = 0.209, but was significantly associated with lower gratitude for males, with b = −0.40, SE = 0.13, t = −3.07, *p* = 0.003, and 95% CI [−0.66, −0.14].

**Figure 1:**
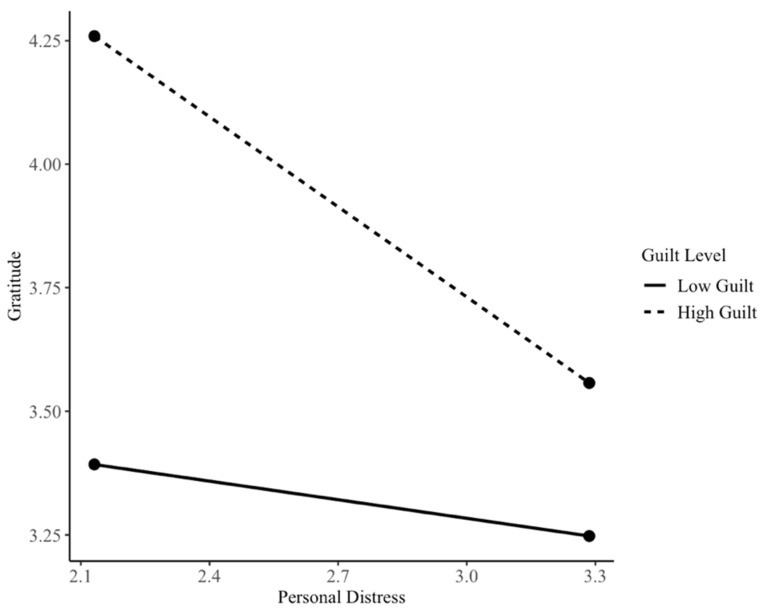
Conditional associations between personal distress and gratitude across levels of guilt among males.

**Figure 2:**
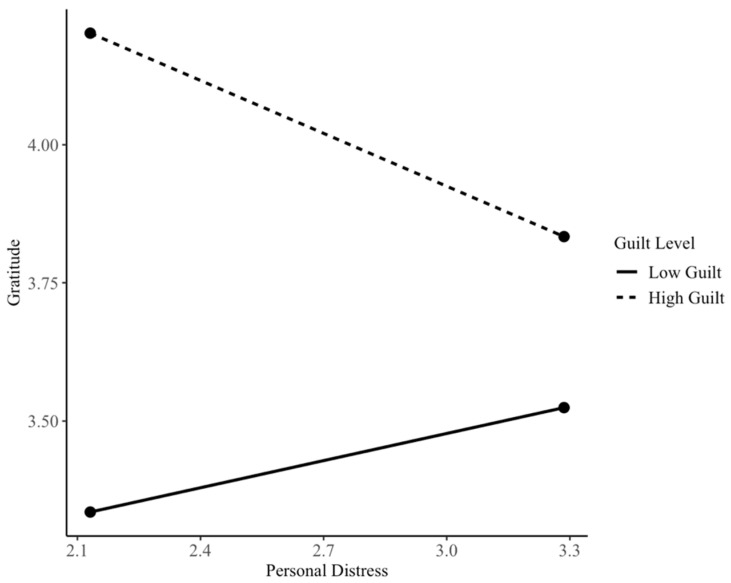
Conditional associations between personal distress and gratitude across levels of guilt among females.

At high levels of guilt, personal distress was significantly associated with lower gratitude for both females, with b = −0.32, SE = 0.11, t = −2.90, *p* = 0.005, and 95% CI [−0.54, −0.10], and males, with b = −0.61, SE = 0.17, t = −3.65, and *p* < 0.001, 95% CI [−0.94, −0.28]. These findings indicate that the negative association between personal distress and gratitude emerges primarily at higher levels of guilt proneness and becomes stronger as guilt increases. (See Figure 1 and Figure 2). Conditional effects were examined separately for descriptive purposes; however, because the personal distress × gender interaction was not statistically significant, these patterns should be interpreted with caution and not as evidence of gender moderation.

## 4. Discussion

The present research examined whether personal distress at Time 1 is associated with gratitude at Time 2 and whether these two variables are moderated by guilt proneness and gender. Personal distress showed a small negative association with gratitude in the regression model including the three predictors; however, this association varied as a function of guilt proneness in the moderation analysis. More specifically, high personal distress was found to relate to low gratitude at high and moderate levels of guilt proneness. Although descriptive conditional effects were found to vary between boys and girls, personal distress and gender interaction effects were found to be statistically non-significant. Therefore, gender should not be interpreted as a supported moderator in the present study.

In the current study, the positive association between guilt and gratitude was found at Time 2. At first, this finding seems to contradict previous studies, which found a negative association between gratitude and guilt [3]. However, this discrepancy may be due to the difference in the conceptualization and measurement of guilt in the two studies. In Watkins et al.’s study, guilt was measured as a situational response to the receipt of a favor and was strongly tied to feelings of obligation and indebtedness, rather than a prosocial empathic response [21].

A key contribution of the present findings is the clarification of the psychological mechanism connecting personal distress and guilt proneness to gratitude. Guilt proneness, in particular, can act as a dispositional lens through which emotionally charged interpersonal experiences are interpreted. When adolescents face personal distress, those with higher guilt proneness are more likely to view this emotional arousal in terms of personal responsibility, self-blame, and obligation instead of mere emotional concern. This understanding aligns with previous research suggesting that guilt-related processes can entail an exaggerated sense of responsibility for others’ well-being [50] and that guilt proneness can be linked to more critical and over-identified reactions to wrongdoing [51].

As predicted by the broaden and build theory [43,52,53], the experience of greater distress was related to lower gratitude in adolescents who also tended to be high in guilt proneness. While the broaden and build theory is primarily concerned with positive emotions, it also suggests that the experience of more negative emotional arousal may narrow attentional and cognitive resources, making it difficult to access feelings of gratitude. In contrast, guilt proneness was positively associated with gratitude when personal distress was relatively low. One way to understand these findings is that, at low levels of personal distress, guilt proneness may be associated with greater awareness of others’ intentions, sacrifices, and support, which could facilitate gratitude. In these circumstances, guilt may facilitate gratitude by shifting attention outward, toward the recognition of support. However, when guilt and high personal distress co-occur, the combination seems to be related to lower gratitude. In these circumstances, guilt may be experienced more as self-blame [54], responsibility, and pressure to repay, which could constrict attention and reduce openness to positive emotions necessary for gratitude. This is consistent with previous findings that indebtedness and guilt are related to pressure and poor well-being [37,55]. More broadly, developmental extensions of broaden and build theory suggest that positive emotions are embedded in social relationships and may contribute to later socioemotional resources over time [56].

These results do not imply that adolescents who are highly distressed and/or guilt-prone take their benefits for granted [34]. Instead, the findings suggest that adolescents differ in how they evaluate their benefits. For example, adolescents who experience high personal distress and guilt may acknowledge that others have sacrificed much for their benefit, and that they should indeed be grateful. However, they may only experience their benefits as debts, and feel pressured and stressed that they have to repay [32]. Moreover, lower gratitude has also been associated with higher levels of neuroticism. Gratitude has been found to be positively related to agreeableness, openness to experience, extraversion, and negatively to neuroticism [57]. Adolescents high in neuroticism experience more frequent and intense experiences of negative affect and distress [58] One possible interpretation is that adolescents who are high in both distress and guilt proneness may overlap with those higher in neuroticism, making them especially susceptible to viewing supportive experiences through the lens of worry, self-criticism, and obligation. From this viewpoint, gratitude in adolescence may be best conceptualized not merely as a dispositional trait, but as an outcome variable influenced by the interplay of emotional (distress, guilt) and personality-related variables. However, this interpretation remains speculative, as personality variables were not directly assessed in the present study. These findings align with broader work suggesting that self-oriented empathic responses (e.g., personal distress) may constrain prosocial tendencies, whereas other-oriented responses (e.g., empathic concern) are more consistently linked to prosocial engagement [59,60].

The conditional effects were explored for both genders separately for descriptive purposes only. Personal distress had a negative association with gratitude at moderate-to-high levels of guilt proneness for boys, while for girls, the pattern emerged at high levels of guilt proneness. However, these results are limited as the interaction between personal distress and gender was not statistically significant. Thus, these results may only suggest potential trends for replication.

## 5. Limitations

A key limitation of the present study is the absence of a baseline measure of gratitude at Time 1. As a result, the analyses do not model change in gratitude over time, and it is not possible to determine whether personal distress and guilt proneness predict intra-individual changes in gratitude. Instead, the findings reflect prospective associations between earlier emotional characteristics and later gratitude. Future research should include repeated assessments of gratitude across multiple time points to allow for autoregressive or growth modeling approaches that can more rigorously examine developmental change.

A second limitation is the gender imbalance in the sample, with more female participants. Therefore, the current findings should be interpreted with caution, and future studies should attempt to replicate the current findings with more gender-equitable samples. Although the sample size was adequate to detect the observed interaction between personal distress and guilt, replication of the study in larger samples is required, especially to explore gender-related patterns. Moreover, all the measures were assessed using self-report questionnaires. Although this is acceptable in the context of the current exploratory study, future studies would benefit from using multi-method designs (e.g., behavioral measures, reports from other people, or experimental designs) to enhance inference. Because all variables were assessed using self-report measures within the same context, the observed associations may be partially influenced by shared method variance, such as response styles or affective state at the time of reporting [61]. In addition, Longitudinal measurement invariance of the gratitude measure could not be evaluated because gratitude was assessed only at Time 2. In addition, the sample was drawn from predominantly Canadian, middle-class backgrounds, which may limit the generalizability of the findings to more diverse cultural contexts.

## 6. Implications and Future Directions

Although the present study did not examine interventions, future research could examine the relation between adolescents’ levels of guilt proneness/personal distress and their reactions to interventions based on gratitude. That is, it could be beneficial for future research to examine whether adolescents’ interpretations related to guilt inform their reactions to interventions based on gratitude. These ideas are purely speculative, however, and need to be tested. Future research could also examine whether these findings generalize to other types of interventions, such as mindfulness-based interventions or those based on gratitude delivered within cultural/religious contexts [62,63].

Future research should explore multiple methods for measuring empathy. Previous studies indicate that empathy assessments in young people differ significantly in what they evaluate and in terms of their reliability, and often they do not correlate with one another [64]. Since self-report questionnaires might only capture a portion of empathy, combining them with behavioral or observational methods could offer a more comprehensive understanding, particularly for specific aspects like personal distress.

## 7. Conclusions

This study examined the association between personal distress and gratitude in adolescents and found that personal distress was associated with lower gratitude among youth high in guilt proneness. This indicates that for these youth, increased personal distress is related to lower levels of gratitude. Most importantly, these findings indicate that lower levels of expressed gratitude do not necessarily imply ingratitude, and personal distress and guilt may impede the capacity to access and profit from gratitude. Some youth may be aware that they “should” be grateful and even report feeling grateful, yet they do not profit emotionally from their gratitude. This may be particularly the case for adolescents, who are high in guilt proneness, as their gratitude may be felt not as appreciation, but as a sense of obligation or pressure to reciprocate. The findings should be considered in light of the limitations of the current research. These limitations include the lack of baseline levels of gratitude and the lack of significant moderation effects for gender. The findings highlight the importance of considering guilt proneness in the study of adolescents’ expressions of gratitude and point to a fruitful avenue for further research in the area.

## Figures and Tables

**Table 1:** Means, standard deviations, and Pearson correlation coefficients.

Variable	M	SD	1	2	3
1. Guilt (Time 1)	4.02	0.58	—		
2. Gratitude (Time 2)	3.68	0.58	0.48 **	—	
3. Personal distress (Time 1)	2.78	0.67	0.12	−0.09	—

Note: ** *p* < 0.01.

## Data Availability

The data are not publicly available due to ethical and privacy restrictions but may be available from the corresponding author upon reasonable request.
